# Heterogeneous expression of ACE2 and TMPRRS2 in mesenchymal stromal cells

**DOI:** 10.1111/jcmm.17048

**Published:** 2021-11-24

**Authors:** Melanie Generali, Debora Kehl, Debora Wanner, Michal J. Okoniewski, Simon P. Hoerstrup, Paolo Cinelli

**Affiliations:** ^1^ Institute for Regenerative Medicine (IREM) Center for Therapy Development and Good Manufacturing Practice University of Zurich Zurich Switzerland; ^2^ Scientific IT Services ETH Zurich ETH Zurich Zürich Switzerland; ^3^ Center for Applied Biotechnology and Molecular Medicine (CABMM) University of Zurich Zurich Switzerland; ^4^ Wyss Zurich University of Zurich and ETH Zurich Zurich Switzerland; ^5^ Department of Trauma Surgery University Hospital Zurich University of Zurich Zurich Switzerland

**Keywords:** adult stem cells, cellular therapy, mesenchymal stromal cells (MSCs), sars‐CoV‐2

## Abstract

The outbreak of COVID‐19 has become a serious public health emergency. The virus targets cells by binding the ACE2 receptor. After infection, the virus triggers in some humans an immune storm containing the release of proinflammatory cytokines and chemokines followed by multiple organ failure. Several vaccines are enrolled, but an effective treatment is still missing. Mesenchymal stem cells (MSCs) have shown to secrete immunomodulatory factors that suppress this cytokine storm. Therefore, MSCs have been suggested as a potential treatment option for COVID‐19. We report here that the ACE2 expression is minimal or nonexistent in MSC derived from three different human tissue sources (adipose tissue, umbilical cord Wharton`s jelly and bone marrow). In contrast, TMPRSS2 that is implicated in SARS‐CoV‐2 entry has been detected in all MSC samples. These results are of particular importance for future MSC‐based cell therapies to treat severe cases after COVID‐19 infection.

## 1 INTRODUCTION

The outbreak of COVID‐19 has become a serious public health emergency. The virus targets cells by binding the angiotensin‐converting enzyme 2 (ACE2) receptor. Viral S‐protein of SARS‐CoV‐2 binds ACE2 and utilizes membrane bound cell transmembrane protease serine 2 (TMPRSS2) to get primed and enter the human cells. ACE2 and TMPRSS2 are expressed on the cell surface of several human cells, for example, alveolar cells and capillary endothelium, but immune cells such as T and B lymphocytes are negative for ACE2.^1^

Even though in most coronavirus disease (COVID‐19) patients, the disease progresses with a mild‐to‐moderate course in ca. In 14% of cases (especially high‐risk patients), the disease can become life‐threatening leading to multiple organ failure and acute respiratory distress syndrome (ARDS) due to cytokine storm syndrome and dysregulated immune response.^2^, ^3^ In this subgroup, the mortality is significantly higher. Many efforts have been invested to develop and deploy safe and effective vaccines. Currently, a number of COVID‐19 vaccines have been rolled out in different countries or are in development.^3^ The advent of new coronavirus variants and the first hints that several vaccines are less effective against them highlight the need of therapeutic treatments especially for high‐risk patients. Among possible treatments, immunoregulatory agents are considered to be valuable. Mesenchymal stromal cells (MSCs) have immunomodulatory properties and regenerative potential and therefore are an excellent candidate for cell therapy and restauration of tissue function. They can be isolated from different sources, including adipose tissue (AD‐MSCs), bone marrow (BM‐MSCs), dental pulp, umbilical cord (UC‐MSCs) and fetal liver.^4^, ^5^ Due to their immunomodulatory properties, MSCs could inhibit the uncontrolled cell‐mediated inflammatory response induced by SARS‐CoV‐2 and eventually reduce acute lung injury. It has been shown that MSCs can improve ARDS based on antimicrobial, anti‐inflammatory, regenerative, antioxidative stress, angiogenic and antifibrotic effects through secretion of anti‐inflammatory factors, such as COX2, IDO, NO, TGF‐β1, PGE2 and HLA‐G5.^3^ MSCs have beneficial effects in ARDS animal models, such as reducing lung injury and maintaining alveolar‐endothelial barrier homeostasis mediated by TGF‐β1, IDO, NO, IL‐1RA, KGF and IL‐10.^6^, ^7^ MSC can be modified in order to optimize and maximize cell survival and beneficial paracrine factors. Application of MSCs overexpressing heme‐oxygenase‐1 (HO‐1) improved survival rate, attenuated lung pathological impairments and suppressed inflammatory reaction in a lipopolysaccharide (LPS)‐induced ARDS rat model.^8^ Furthermore, inhibition of (repressor/activator protein) Rap1 in BM‐MSCs decreased nuclear factor‐kappa B (NF‐*κ*B) sensitivity to stress‐induced proinflammatory cytokine production and reduced apoptosis.^9^, ^10^

Several clinical trials have preliminarily demonstrated the safety and efficacy of intravenous MSCs in patients with COVID‐19‐related lung diseases.^3^, ^11^, ^12^ Conclusive interpretation of these clinical trials is difficult due to the unknown heterogeneity of the MSCs used. Recently, intravenous administration of MSCs of unknown tissue origin to seven patients with different severities of ARDS resulting from COVID‐19 showed in all patients an improvement after two days upon injection linked to an increase of circulating lymphocytes and CXCR3‐negative regulated T cells and dendritic cells suggesting a switch from a proinflammatory to an anti‐inflammatory state.^11^ In another trial, double intravenous injections of 100 ±  20 × 10^6^ UC‐MSCs (subepithelial lining of an UC) were correlated with improvement of patient survival and a significant decrease of the cytokine storm.^12^ The absence of serious adverse events directly related to UC‐MSCs infusions suggest the potential safe use of MSCs for COVID‐19 cell therapy.^12^ Nevertheless, MSCs are highly heterogeneous, pleiotropic and sensitive to different microenvironments and secrete biologically active substances responsively. This characteristic has an impact on their differentiation ability and is an important limitation for their use in therapeutic applications.

Currently available data regarding the expression of ACE2 and TMPRSS2 in MSCs are discordant. Desterke et al. analysed available transcriptome datasets and concluded that both genes are significantly higher expressed in AD‐MSCs and BM‐MSCs compared to MSCs isolated from the umbilical cord or placenta.^13^ In contrast, another study by Avanzini and coworkers showed the absence of expression of ACE2 and TMPRSS2 in MSCs derived from five different tissue sources (amniotic membrane of placenta, cord blood, cord tissue, adipose tissue and bone marrow).^14^ The same study also showed that human MSCs are not permissive to SARS‐CoV2 infection,^14^ which is a prerequisite for their safe therapeutic use for treating COVID‐19.

The aim of the present work was to evaluate whether human MSCs from different tissue sources (AD‐MSCs, umbilical cord Wharton's jelly MSCs (WJ‐MSCs) and BM‐MSCs) express ACE2, TMPRSS2 and additional proviral and antiviral associated genes.

## 2 MATERIAL AND METHODS

### 2.1 Cell culture

Isolation procedure and MSC specific characterisation were performed as described (Kehl et al.).^4^ All MSCs were cultured in the same media: Dulbecco's modified Eagle's medium (DMEM) (Sigma) supplemented with 10% of fetal bovine serum (FBS) (Gibco, Life technologies), 1% of antibiotics (100 × penicillin, 100 × streptomycin) (Merck) and 1% L‐glutamine 200 mM (ThermoFisher Scientific). Medium was changed every 3 days, and cells were passaged with 1× Accutase (Gibco, Life technologies) for 5 min at 37 °C when cells were about 80% confluent. Cells were incubated at 37 °C in an atmosphere with 95% humidity and 5% CO_2_.

Table 1 displays an overview of all MSC donor characteristics.

**TABLE 1:** MSC donor characteristics

Sample Name	Cell Source	Sex	Year of Birth	Year of Isolation	Passage used
AD‐MSC PL2	Adipose Tissue	Female	1970	2014	P9
AD‐MSC PL5	Adipose Tissue	Female	1982	2015	P9
AD‐MSC PL6	Adipose Tissue	Female	1971	2015	P9
AD‐MSC PL7	Adipose Tissue	Female	1942	2015	P9
AD‐MSC PL8	Adipose Tissue	Female	1932	2015	P9
BM‐MSC PL1	Bone Marrow	Male	1942	2016	P4
BM‐MSC PL2	Bone Marrow	Female	1936	2016	P6
BM‐MSC PL5	Bone Marrow	Female	1950	2016	P3
BM‐MSC PL7	Bone Marrow	Female	1941	2016	P5
BM‐MSC PL8	Bone Marrow	Female	1956	2016	P4
WJ‐MSC PL1	Umbilical Cord (Wharton`s Jelly)	Female (Child)	1980 (mother)	2014	P6
WJ‐MSC PL2	Umbilical Cord (Wharton`s Jelly)	Male (Child)	1980 (mother)	2014	P6
WJ‐MSC PL4	Umbilical Cord (Wharton`s Jelly)	Male (Child)	1971 (mother)	2014	P5
WJ‐MSC PL5	Umbilical Cord (Wharton`s Jelly)	Female (Child)	1972 (mother)	2014	P5
WJ‐MSC PL6	Umbilical Cord (Wharton`s Jelly)	Male (Child)	1985 (mother)	2015	P6

### 2.2 RNA Extraction

Total RNA was obtained using the RNeasy Mini Kit (Qiagen) and reverse‐transcribed to cDNA using iScript cDNA Synthesis Kit (Bio‐Rad) according to the manufacturer's protocol. qPCR was performed using iTaq Universal SYBR Green Supermix (Bio‐Rad) and standard conditions on a QuantStudio 7 flex real‐time PCR system (Applied Biosystems). Primers are listed in Table 2. The gene expression level analysis was normalized versus GAPDH and conducted in triplicate.

**TABLE 2:** List of primers

Target	Forward primer	Reverse primer
ACE2	GGGATCAGAGATCGGAAGAAGAAA	AGGAGGTCTGAACATCATCAGTG
GAPDH	ACCACAGTCCATGCCATCAC	TCCACCCTGTTGCTGTA
TMPRSS2	ACTCTGGAAGTTCATGGGCAG	TGAAGTTTGGTCCGTAGAGGC

### 2.3 Western blotting

For western blot, cells were lysed with RIPA buffer (50 mM Tris‐HCl pH 7.6, 150 mM NaCl, 1% NP40, 0.1% SDS, 0.5% sodium deoxycholate, 2 mM EDTA) supplemented with 1 tablet EDTA‐free protease inhibitor cocktail/50 ml (#04693159001, Sigma). Protein concentration was measured with Pierce BCA protein Assay Kit (Thermo Scientific). Proteins were identified by SDS‐PAGE and western blotting using antibodies reported in Tables 3 and 4.

**TABLE 3:** Antibodies for Western Blot and IHC: Primary antibodies

Antibody anti‐human	Host	Company	Dilution	Number	Application
ACE2	rabbit	GeneTex, USA	1:50	GTX01160	WB
ACE2	rabbit	Abcam, UK	1:1000	Ab108252	IHC
GAPDH	mouse	Meridian, USA	1:5000	H86504 M	WB
TMPRSS2	rabbit	GeneTex, USA	1:100 (IHC) 1:500 (WB)	GTX100743	IHC, WB

**TABLE 4:** Secondary antibodies for Western Blot and IHC

Antibody	Dilution	Company	Number	Application
HRP goat anti mouse	1:2000	Jackson, USA	115–035–166	WB
HRP goat anti rabbit	1:2000	Jackson, USA	111–035–144	WB
anti rabbit Alexa Fluor 594	1:1000	Invitrogen, USA	11037	IHC

Protein bands were detected with the ImageQuant LAS 4000 (GE Healthcare Life Sciences), using Pierce™ ECL Western Blotting Substrate (#32106, ThermoFisher Scientific) or with SuperSignal West Femto Maximum Sensitivity Substrate (ThermoFisher Scientific). Image analysis was carried out in ImageJ. Signals were normalized to GAPDH probed on the same blot.

### 2.4 Immunohistochemistry

Cells were fixed with 4% paraformaldehyde and incubated over night at 4 °C with primary antibodies (Table 3). Secondary antibodies were incubated for 1 h (Table 4), and pictures were taken and analysed with XX fluorescence microscope.

### 2.5 RNA sequencing

Strand‐specific mRNA‐seq libraries for the Illumina platform were generated and sequenced by Eurofins GmbH.

### 2.6 Bioinformatic processing and analysis

The Illumina reads of 15 samples have been processed in a Snakemake workflow to obtain a table with per‐gene counts for each sample. The workflow included trimmomatic (v0.35), alignment with STAR (v2.7.0) to hg38, sorting and indexing the alignment files with samtools (v1.9), and counting with featureCounts from subread package (v1.5), using Ensembl human annotations GTF file (v100) and parameters for reverse‐stranded (Illumina TruSeq) libraries. The count vectors for all the samples have been combined into a count table. The count table has been processed in the secondary (statistical) analysis with R scripts using DESeq2 (v2_1.30), in particular, binomial generalized log‐linear model with contrast tests. It resulted in lists of genes ranked for differential expression by p‐value and used Benjamini‐Hochberg adjusted p‐value as the estimate of the false discovery rate. The quality control and sample consistency have been checked then with PCA, using R package FactoMiner (v2.3), while log2 count values have been plotted onto heatmaps using pheatmap (v1.0.12).

## 3 RESULTS

**FIGURE 1:**
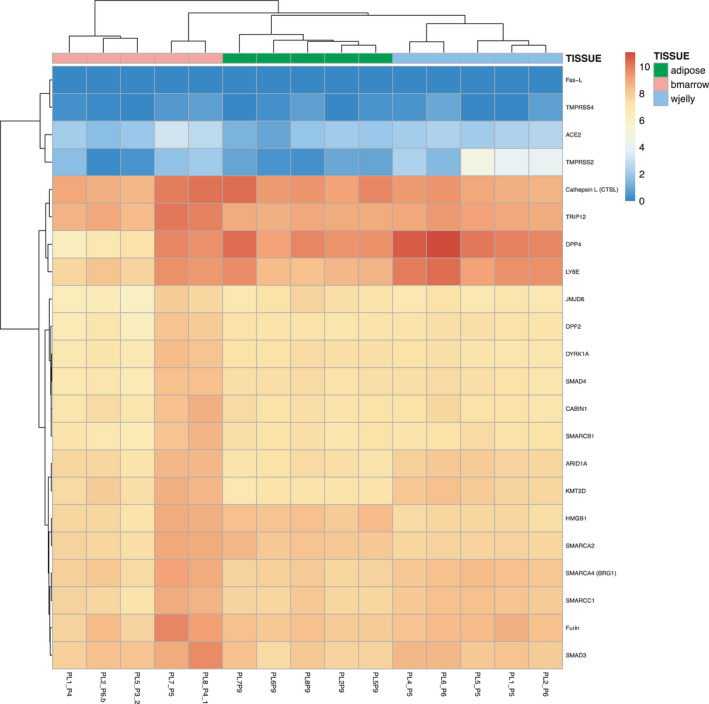
Heat map of RNA‐Seq transcriptome analysis for 22 selected genes. Log2 count values have been plotted onto heatmaps. The heatmap was drawn using pheatmap (pretty heatmaps) library using standard hclust R function for hierarchical clustering with euclidean distance measure and “complete” agglomeration method. Results of five MSCs donors per tissue source are displayed in green for AD‐MSC, in pink for BM‐MSCs and in blue for WJ‐MSCs

We have performed RNA sequencing on human MSCs isolated from three different tissue sources (Suppl. Material Table 1: AD‐MSCs *n* = 5, WJ‐MSCs *n* = 5 and BM‐MSCs *n* = 5) and assessed the expression levels of ACE2, TMPRSS2 and additional proviral and antiviral associated genes (Figure 1).

ACE2 as well as Fas‐L (Fas‐Ligand) and TMPRSS4 (transmembrane serine protease 4), two additional genes involved in the infection process of SARS‐CoV‐2 were not expressed neither in AD‐MSCs, WJ‐MSCs nor in BM‐MSCs. Of interest, dipeptidyl peptidase 4 (DPP4), which, like ACE2, has been shown to serve as a binding partner for corona‐like viruses to enter host immune cells,^15^ was found to be higher in the WJ‐MSCs in comparison with BM‐MSCs (age above 55) (Figure 1). This is in contrast to previous studies claiming that DPP4 activity is higher in older individuals and DPP4 upregulation may be a determinant of COVID‐19 disease severity.^16^ TMPRSS2 expression was generally low in AD‐MSCs and BM‐MSCs, but in 3 out of 5 WJ‐MSCs expression was higher.

**FIGURE 2:**
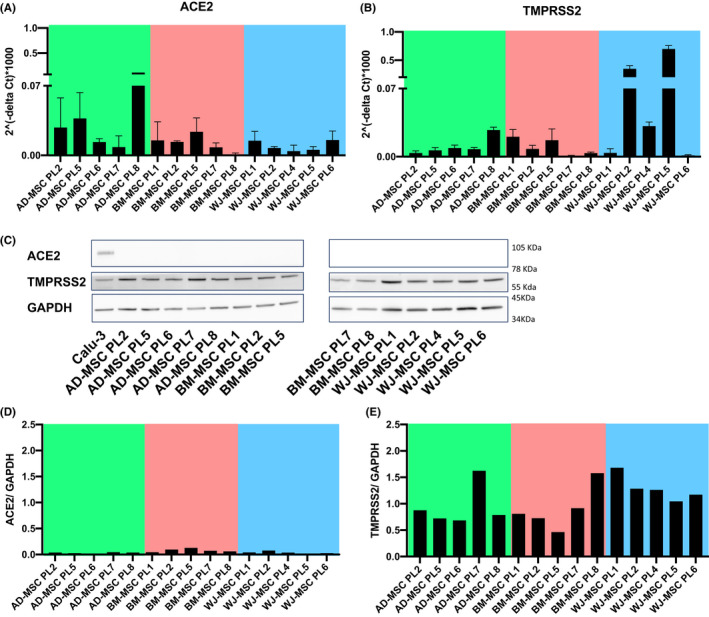
ACE2 and TMPRSS2 expression in human AD‐MSCs, BM‐MSCs and WJ‐MSCs. Relative expression by quantitative reverse transcription PCR (RT‐qPCR) of (A) ACE2 and (B) TMPRSS2 mRNA levels in AD‐MSCs (n = 5), BM‐MSCs (n = 5), and WJ‐MSCs (n = 5). Data are normalized with GAPDH. C, Representative Western blot of ACE2 and TMPRSS2 of the three different MSC types. D, Quantification showed a nearly no detectable expression of ACE2. E, Heterogenous expression of TMPRS2 in MSCs. Cell line Calu‐3 was used as a positive control. GAPDH was used as loading control for cell lysates

Expression of both ACE2 and TMPRSS2 was further validated by quantitative real‐time PCR (Figure 2A,2B), western blotting (Figure 2C–E) and immunohistochemistry (IHC) (Figure S1). ACE2 protein was absent in all MSC lysates, whereas TMPRSS2 expression was present in all three tissue sources being in WJ‐MSCs higher than in AD‐MSCs or BM‐MSCs.

## 4 DISCUSSION

Our data show a heterogeneous expression of ACE2 and TMPRRS2 in MSCs between donors and tissue source. Individual characteristics and age‐related variability have to be taken into consideration when planning to use MSCs for COVID‐19 treatment purposes. A recent work highlighted the importance of age showing that ACE2^+^ cells were highly enriched in lung tissues of children and gradually decreased with increased age.^17^ These findings underline that disease severity between younger and older patients does not mainly depend on the ACE2 expression level. Furthermore, ACE2 and TMPRSS2 expression levels were detected in heterogeneous cell types, such as hepatocyte/endodermal, epithelial, goblet, proximal tubule progenitor, fetal enterocyte and enterocyte cells and various tissue types, including the intestine, duodenum, gallbladder, kidney, ileum, adrenal gland and transverse colon.^18^ Importantly, only stromal cells showed a significant age‐related change.

Human‐induced pluripotent stem cell (iPSC)‐derived MSCs (iMSCs) represent an alternative MSC source. Interestingly, iMSCs have been shown to suppress lung inflammation and reduce oxidative stress, which play an important role in SARS‐Cov‐2 infections.^19^, ^20^ Compared to BM‐MSCs, iMSC displayed poor immunogenicity after IFN stimulation in vitro and higher cell retention, less inflammation and better efficacy in an immune humanized ischaemic mice.^21^ These cells may have better potential for clinical applications in allogenic transplantation.

## 5 CONCLUSION

Tissues and organs expressing the ACE2 receptor are susceptible to the SARS‐CoV‐2 infection. Our data demonstrate that MSCs derived from different human tissues do not express ACE2 but TMPRSS2. Currently, there are only few sufficient clinical data about the potential clinical efficacy of MSC to treat patients with severe COVID‐19 disease, but clinical trials are urgently needed. Our findings emphasize a detailed characterisation before administration of these cells, pointing out tissue‐specific as well as donor‐specific variability.

## CONFLICT OF INTEREST

The authors declared no potential conflicts of interest.

## AUTHOR CONTRIBUTION

**Melanie Generali:** Conceptualization (lead); Funding acquisition (equal); Project administration (lead); Supervision (lead); Writing‐original draft (lead). **Debora Kehl:** Conceptualization (equal); Funding acquisition (equal); Writing‐review & editing (supporting). **Debora Wanner:** Methodology (lead). **Michal J. Okoniewski:** Data curation (lead); Software (lead); Writing‐review & editing (supporting). **Simon Philipp Hoerstrup:** Funding acquisition (equal); Resources (equal); Writing‐review & editing (equal). **Paolo Cinelli:** Formal analysis (equal); Funding acquisition (equal); Supervision (equal); Writing‐review & editing (lead).

## ETHICAL APPROVAL

MSCs were obtained from human adipose tissue, umbilical cord (Wharton`s jelly derived (WJSCs)) and bone marrow aspirate upon written informed consent of the donors, following the guidelines approved by the cantonal ethics commission (Kantonale Ethik Kommission (KEK)) Zurich Switzerland (KEK‐ZH‐2010–0476, KEK‐ZH‐2009–0095).

## CONSENT FOR PUBLICATION

Not applicable.

## Supporting information

Fig S1

## Data Availability

RNA sequencing data that support the findings of this study are available on request from the corresponding author [M.G.].
