# Supplementary material for: Heterogeneous expression of ACE2 and TMPRRS2 in mesenchymal stromal cells
Source: J Cell Mol Med. 2021 Nov 24;26(1):228–34. doi: 10.1111/jcmm.17048 (PMC8742235; doi:10.1111/jcmm.17048)
Supplement: Supplementary file 1 — Fig S1 [file JCMM-26-228-s001.docx]

**Supplementary Data**

**Figure 1**

**Figure 1:** Immunostaining for AD-MSC (PL8), BM-MSC (PL5) and WJ-MSC (PL1). Calu-3 was used as a positive control. Following markers were tested: ACE2 and TMPRRS2.

(Scale bars: 100 μm.)
